# Mutational analysis of Rift Valley fever phlebovirus nucleocapsid protein indicates novel conserved, functional amino acids

**DOI:** 10.1371/journal.pntd.0006155

**Published:** 2017-12-21

**Authors:** Timothy J. Mottram, Ping Li, Isabelle Dietrich, Xiaohong Shi, Benjamin Brennan, Margus Varjak, Alain Kohl

**Affiliations:** MRC-University of Glasgow Centre for Virus Research, Glasgow, Scotland, United Kingdom; New York University, UNITED STATES

## Abstract

Rift Valley fever phlebovirus (RVFV; *Phenuiviridae*, *Phlebovirus*) is an important mosquito-borne pathogen of both humans and ruminants. The RVFV genome is composed of tripartite, single stranded, negative or ambisense RNAs. The small (S) segment encodes both the nucleocapsid protein (N) and the non-structural protein (NSs). The N protein is responsible for the formation of the viral ribonucleoprotein (RNP) complexes, which are essential in the virus life cycle and for the transcription and replication of the viral genome. There is currently limited knowledge surrounding the roles of the RVFV nucleocapsid protein in viral infection other than its key functions: N protein multimerisation, encapsidation of the RNA genome and interactions with the RNA-dependent RNA polymerase, L. By bioinformatic comparison of the N sequences of fourteen phleboviruses, mutational analysis, minigenome assays and packaging assays, we have further characterised the RVFV N protein. Amino acids P11 and F149 in RVFV N play an essential role in the function of RNPs and are neither associated with N protein multimerisation nor known nucleocapsid protein functions and may have additional roles in the virus life cycle. Amino acid Y30 exhibited increased minigenome activity despite reduced RNA binding capacity. Additionally, we have determined that the N-terminal arm of N protein is not involved in N-L interactions. Elucidating the fundamental processes that involve the nucleocapsid protein will add to our understanding of this important viral protein and may influence future studies in the development of novel antiviral strategies.

## Author summary

Rift Valley fever phlebovirus (RVFV) remains an ongoing serious socio-economic threat, and with a lack of widely available licensed vaccines and treatments it is increasingly important to understand the underlying biology behind RVFV viral replication. Re-analysis of RVFV nucleocapsid, a key protein within the RVFV replication cycle has led to the characterisation of previously unidentified conserved amino acids. This study increases our understanding of the nucleocapsid protein which may play an important role in the future development of vaccine and antiviral therapies.

## Introduction

RVFV is a member of the *Phlebovirus* genus of the *Phenuiviridae* family of viruses, in the *Bunyavirales* order [1]. The *Phlebovirus* genus can be broadly split into two distinct groups. One group pertains to the viruses transmitted by mosquitos or sandflies, commonly referred to as the Phlebotomine group, of which RVFV is a member. The second group pertains to viruses transmitted primarily by ticks [2]. The RVFV genome consists of two negative and one ambisense RNA segment. The large (L) segment encodes the RNA dependent RNA polymerase. The medium (M) segment encodes two non-structural proteins (p78 and NSm) and two glycoproteins, Gn and Gc [3]. The small (S) segment encodes the nucleocapsid protein (N) and the non-structural protein (NSs) using an ambisense coding strategy [4]. Each genome segment is flanked by untranslated regions (UTRs) that are involved in regulating transcription and replication of the genome segments. Due to terminal sequence complementarity, the 3’ and 5’ UTRs base pair and generate panhandle structures resulting in the recruitment of the viral polymerase (L), thus allowing the formation of viral ribonucleoprotein complexes (RNPs) [5, 6] as is the case for all bunyaviruses [2, 7, 8]. RVFV N protein is a key protein within the RVFV proteome. It is characterised by a protruding N-terminal arm, an RNA binding cleft and a multimerisation groove. It has several essential functions that allow the replication and transcription of the viral genome segments. The N proteins of viruses belonging to the *Bunyavirales* order have been shown to function to encapsidate the viral genome which protects the genetic information from harsh conditions found in the intracellular environment, such as RNase degradative enzymes [9]. This encapsidation function of the N protein and the formation of the viral RNP complex allow the binding of the RNA-dependent RNA-polymerase thereby allowing transcription and replication to take place. Furthermore, N protein forms multimeric structures in infected cells [10–12]; in the case of RVFV the binding of the N-terminal arm to adjacent N monomers results in the formation of ring-shaped oligomers and allows the creation of filamentous RNPs required for replication of the viral genome [10].

N proteins of other families in the *Bunyavirales* order have also been investigated and have added to our understanding of N protein function. For the related genus *Orthohantavirus*, the N protein is thought to have RNA chaperoning activity where it dissociates the viral RNA duplexes, allowing sequestration of the 5’ end of the RNA and the binding of the L protein [13, 14]. Additionally, Sin Nombre virus (of the genus *Hantavirus*) N has been shown to bind mRNA caps, an essential process in cap-snatching [15]. The *Bunyavirales* order is very broad and viral N protein structures from different families are often unrelated, as evidenced by the Crimean-Congo haemorrhagic fever orthonairovirus (CCHFV) N protein. CCHFV is part of the *Orthonairovirus* genus within the *Nairoviridae* family and has a distinct N protein structure more closely related with the *Arenaviridae* family compared to other members of the *Bunyavirales* [16]. In the case of the *Peribunyaviridae*, the order prototype bunyavirus Bunyamwera orthobunyavirus (BUNV) nucleocapsid protein has also been shown to encapsidate the viral genome and carries out largely the same functions as demonstrated for phleboviruses. A previous mutagenesis study carried out using BUNV identified several residues that impact the replication efficiency of the virus [17].

Several studies examining RNA binding have identified residues within the core of the RVFV N protein that are essential for the formation of viral RNPs [10, 18]. The bound RNA interacts with 18 conserved residues within the protein core and the hinge region between the N-terminal arm and the core. In particular, amino acid Y30 is located at the hinge region of the N-terminal arm and has been shown to stack with the 5’ most RNA base when binding RNA [19]. Residues R64D, K67D and K74D were predicted to form the RNA binding cleft and a triple substitution mutant resulted in the loss of RNA binding [10]. The formation of RNPs requires N monomers to form hexameric ring structures through the binding of the N-terminal arm of one monomer to an adjacent monomers oligomerisation groove [10]. Residues Y3, L7, I9, P11, V16, I21, W23, V25, F28 and Y30 present on the N terminal arm of the RVFV N protein are predicted to interact with the oligomerisation groove. Disruption of the oligomerisation groove prevents the formation of higher order N protein structures and thus stops the formation of the viral RNPs [20]. N protein also has a dimeric closed conformational state which is predicted to occur in the absence of viral or host RNA [10]. In the presence of RNA, the conformation opens the N-terminal arm allowing the binding to adjacent subunits forming higher order structures [10]. Several RVFV N protein studies have identified the functions of named amino acid residues and these are summarized in Table 1 [10, 18–20]. These were found to be involved in either N multimerisation or RNA binding, and were used as a reference for selecting the mutants in this study that have been previously unexplored. This study aimed to widen RVFV N protein research by informing on a number of non-characterized, yet conserved amino acids found across multiple members of the *Phlebovirus* genus. As we believed these to be essential nucleocapsid residues with regards to involvement in important protein-protein interactions and other functions, we generated a panel of uncharacterised N protein mutants based on conservation data. These mutant proteins were investigated by utilising minigenome assays, packaging assays as well as biochemical techniques to assess their relevance in the RVFV life cycle. This analysis revealed that there are still fundamental underlying questions regarding the formation of phlebovirus RNP complexes, in particular, regarding residues P11 and F149 that confer loss of function that were not previously described. Our data add to the understanding of RVFV N and the role(s) of individual conserved amino acids in replication.

**Table 1:** Summary of known RVFV N protein functions. Compiled predicted and known functions of RVFV N protein from studies focused on uncovering the RNA binding properties of RVFV N. Functional information was determined through varied methods, including analysis of RVFV N crystal structure and mutagenesis studies [10, 18, 19].

Residue	Function
Met1	Contacts Trp125 dimer interface [18]
Tyr3	Project from N terminal arm—interact with hydrophobic groove [10]
Tyr4	Observed loss of dimer formation (destabilisation of helix a1) [18]
Gln5	Contacts Trp125 dimer interface [18]
Leu7	Project from N terminal arm—interact with hydrophobic groove [10]
Ile9	Contacts Trp125 dimer interface, Project from N terminal arm—interact with hydrophobic groove [10, 18]
Phe11	Observed loss of dimer formation (destabilisation of helix a1), Project from N terminal arm—interact with hydrophobic groove [10, 18]
Ala12	Intersubunit van der waals contacts [18]
Val16	Project from N terminal arm—interact with hydrophobic groove [10]
Ile21	Project from N terminal arm—interact with hydrophobic groove [10]
Trp24	Project from N terminal arm—interact with hydrophobic groove [10]
Val25	Project from N terminal arm—interact with hydrophobic groove [10]
Phe28	Project from N terminal arm—interact with hydrophobic groove [10]
Tyr30	Project from N terminal arm—interact with hydrophobic groove, Hinge region stacks with 5'most base in RNA binding (base 1) [10, 19]
Phe33	"Back pocket" of RNA binding slot interacts with base 2 [19]
Arg64	Predicted RNA binding cleft, loss of RNA binding in triple mutant* [10]
Gly65	Interacts with base 5 in narrow pocket [19]
Lys67	Predicted RNA binding cleft, loss of RNA binding in triple mutant* [10]
Lys74	Predicted RNA binding cleft, loss of RNA binding in triple mutant* [10]
Ala109	Lines RNA binding slot interacts with base 3 and 4 [19]
Ala110	Lines RNA binding slot interacts with base 3 and 4 [19]
Val120	Intersubunit van der Waals contacts [18]
Val121	Intersubunit van der Waals contacts [18]
Glu124	Intersubunit van der Waals contacts [18]
Trp125	Contacts Met1, Gln5, Ile9 and Trp125 of second monomer. Critical for dimer formation [18]
Leu126	Interacts with base 5 in narrow pocket [19]
Pro127	Interacts with base 5 in narrow pocket [19]
Thr131	Intersubunit van der Waals contacts [18]
Pro147	Lines RNA binding slot interacts with base 3 and 4 [19]
Phe176	Interacts with base 5 in narrow pocket [19]
Arg178	Forms salt bridge with Ala245 [18]
Ile180	Lines RNA binding slot interacts with base 3 and 4 [19]
Pro199	Lines RNA binding slot interacts with base 3 and 4 [19]
Ala202	Lines RNA binding slot interacts with base 3 and 4 [19]
Ala245	Forms salt bridge with Arg178 [18]

## Materials and methods

### Cells

BSR-T7/5 cells [21] (obtained from K.-K. Conzelmann, Ludwig-Maxmilians-Universität München, Germany) were grown in Glasgow Minimum Essential Medium (GMEM; Gibco) supplemented with 10% fetal calf serum (FCS), 1% penicillin/streptomycin and the selection agent G418 at 37°C with 5% CO_2_. BSR-T7/5 CL21 cells were generated during this study through dilution cloning of BSR-T7/5 cells. This clone showed increased T7 RNA polymerase-mediated gene expression.

### Plasmids

Plasmids pTM1-N, pTM1-L, pTVT7-GM:hRen and pTM1-FF-Luc have been described previously [6]. pTM1-N and pTM1-L plasmids contain the RVFV MP12 strain N and L open reading frames (ORF) under the control of a T7 promoter. pTM1-L3V5 has been previously described [22] and contains a V5 tagged epitope within the L ORF. pTVT7-GM:hRen contains the humanised *Rluc* ORF in place of the M polyprotein precursor ORF (still flanked by M UTRs) in antisense orientation under the control of a T7 promoter. pTM1-FF-Luc expresses *Photinus* (*FFluc*) luciferase and was used as a transfection control in the minigenome assay. Plasmid p14-N contains the RVFV MP12 strain N ORF with N-terminal 6His-tag attached for bacterial expression and purification. The virus-like particle assays were carried out using the pTM1-based plasmids above, with the addition of pTM1-M.

### Sequence alignments

Sequence alignments were performed using CLC-Genomic Workbench using the following sequences: Punta Toro phlebovirus (Adames) (ABD92922.1); sandfly fever Naples phlebovirus (Namru) (AEL29662.1); Candiru phlebovirus (YP_004347994.; Ixcanal phlebovirus (CA_Ar_170897) (AEB70980.1); RVFV (MP12) (AAF00694.1); Salehabad phlebovirus (I-81) (AGA82743.); Granada phlebovirus (GR25) (ADO17682.1; Toscana phlebovirus (ELB) (ABQ23554.1; sandfly fever Turkey phlebovirus (Izmir19) (AEB97382.1); Uukuniemi phlebovirus (UUKV) (S23) (AAA47958.1); Lone Star phlebovirus (TMA1381) (YP_008003509.; Bhanja phlebovirus (M3811) (AFO66274.1); SFTS phlebovirus (AHZ/China/2011) (AFJ15066.1); Heartland phlebovirus (TN) (AIF75091.1).

### Site-directed mutagenesis

A QuickChange II Site-Directed Mutagenesis Kit (Agilent Technologies) was used for primer-specific mutagenesis of the pTM1-N plasmid N ORF as per the manufacturer’s instructions. The mutants delN1-14, delN1-31, F11A, Y30A, D34A, F149A and N181A were generated from RVFV MP12 pTM1-N and p14-N.

### Minigenome assays

BSR-T7/5 cells were plated at a density of 1x10^5^ cells per well in a 24 well plate. After 24 hours, cells were transfected with 0.2 μg pTM1-L, 0.5 μg pTVT7-GM:hRen, 25 ng pTM1-FF-Luc and 1 μg pTM1-N or one of the pTM1-N mutant clones using Lipofectamine 2000 transfection reagent (Life Technologies) according to manufacturer’s instructions. At 24 h post-transfection cells were lysed in 100 μl passive lysis buffer (Promega). *Rluc* and *FFluc* activities were measured according to the manufacturers’ instructions on a Promega Glomax multidetection system using Dual-Luciferase Reporter Assay Systems reagents (Promega).

### Virus-like particle formation assay

BSR-T7/5 cells were plated at a density of 2 x10^5^ cells per well. After 24 h, cells were transfected with 0.25 μg pTM1-L, 0.5 μg pTVT7-GM:hRen, 0.5 μg pTM1-M, 25 ng pTM1-FF-Luc and 0.5 μg pTM1-N or one of the pTM1-N mutant clones using LT1 transfection reagent (Life Technologies) according to manufacturer’s instructions. At 48 hours post-transfection, the supernatant was removed and treated with 2ul Benzonase nuclease (Merck Millipore) for 4 h at 37°C. Of the treated supernatant, 160 μl was subsequently added to a 12-well plate of BSR-T7/5 cells pre-transfected 24 h prior with 0.5 μg pTM1-N and 0.5 μg pTM1-L. At 24 h post VLP infection, cells were lysed in 200 μl passive lysis buffer (Promega). *Rluc* and *FFluc* activities were measured as described above.

### Immunoblotting

Cell lysates were separated on Bolt 4–12% Bis-Tris Plus (Novex) gradient gels. Proteins were transferred onto 0.45 μm nitrocellulose membranes (Amersham Protran) using Trans-blot SD cell (Bio-Rad). The membrane was blocked using PBS containing 0.1% Tween 20 and 5% Marvel milk powder for 1 h at room temperature. The membrane was incubated with either anti-RVFV N polyclonal rabbit antibody (1:5000) [23] or beta-actin rabbit monoclonal antibody (1:1000; Cell Signalling Technology) overnight at 4°C. A horseradish peroxidase (HRP) anti-rabbit secondary antibody (1:1000) was then applied to the membrane. Pierce ECL Western Blotting Substrate (Thermo-Scientific) followed by visualisation on a Biorad ChemiDoc system.

### Protein expression and purification in *Escherichia coli*

The p14-N protein expression plasmids for each mutant N protein were transformed into Rosetta2 (DE3) cells (Novagen). Subsequent colonies were grown in LB Broth to OD0.6–0.8 before inducing with 1 mM IPTG, and the cells were cultured at 20°C for overnight expression. The bacteria were pelleted for 30 min at 3600 x g. The pellet was resuspended in 12 ml B-PER Complete Bacterial Protein Extraction Reagent (Thermo Scientific) supplemented with EDTA-free Protease Inhibitor Cocktail (Thermo Scientific) and rotated at room temperature for 1 h. After lysis, 50 mM Tris pH8.0, 20 mM Imidazole, 0.3 M NaCl, 10% glycerol was added to the lysate. The lysate was centrifuged at 4400 x g for 30 min at 4°C and the supernatant was collected. For each sample, 900 μl of the Ni-NTA resin (Thermo Scientific) was equilibrated with 50 mM Tris pH8.0, 20 mM imidazole, 0.3 M NaCl, 10% glycerol. The clarified supernatants were added to equilibrated Ni-NTA resin tube and incubated at 4°C with gentle rotation for 30 min. The samples were then spun at 2000 x g for 1 min and the supernatant removed. The Ni-NTA resin was washed with buffer containing 50 mM Tris pH8.0, 40 mM imidazole, 0.3 M NaCl, 10% glycerol three times. The protein was eluted with 1.5 ml of elution buffer containing 10 mM Tris pH 8.0, 200 mM imidazole, 0.3 M NaCl and 10% glycerol. Vivaspin 500 Centrifugal Concentrator (Sartorius) was used to exchange the elution buffer for protein storage buffer (10 mM Tris pH8.0, 150 mM NaCl, 10% glycerol) and protein samples were stored at -80°C. Purified protein samples were visualised on Bolt 4–12% Bis-Tris Plus (Novex) gradient gels, stained with InstantBlue Protein Stain (Expedeon).

### DSP crosslinking assay

The purified mutant RVFV N proteins were concentrated to 0.2 μg/μl in 25 μl PBS/glycerol using Vivaspin centrifugal concentrators (Sartorius). Dithiobis(succinimidyl propionate)(DSP)-Lomants reagent (Thermo Scientific) was added to a final concentration of 1 mM and gently mixed, then incubated at 20°C for 40 min. The reaction was stopped by directly adding protein loading buffer under non-reducing conditions and then analysed on a 4–12% Bis-Tris plus (Novex) gradient gel.

### RNA binding assay

Recombinant parental virus (rMP12) and mutant N proteins purified by Ni-NTA were examined for RNA-binding activity. 10 μg of purified protein was mixed with 2x RNA gel loading buffer (Thermo Scientific) containing 95% formamide. The RNA was separated on the 2% TopVision Agarose gel (Thermo Scientific) in TBE buffer stained with Gel Red (Biotium) and visualised by UV.

### Immunoprecipitation

BSRT-7/5 CL21 cells were seeded into 6-well plates at 4.8 x10^5^ cells per well. Each well was transfected with 3 μg pTM1-N or a mutant pTM1-N and 2 μg pTM1-L3V5 expression plasmids. After 24 h, cells are lysed with lysis buffer containing 20 mM Tris pH 7.5, 150 mM NaCl, 5 mM MgCl_2_, 0.5% Triton X-100, and Halt Protease inhibitor cocktail (Thermo Scientific) and 10 μg RNase (Thermo Scientific). The lysate was kept on ice for 20 min then centrifuged at 16000 x g for 20 min to pellet the cellular debris. Protein A beads were bound with anti-V5 antibody (#27671, Abcam). Lysates were rotated with beads for 2 h, after which, the beads were washed three times with lysis buffer and eluted by heating the sample to 90°C in LDS Sample buffer and 2-mercaptoethanol reducing agent (Thermo Scientific). Samples were analysed by western blotting with primary anti-RVFV N antibody (1:5000) or anti-V5 antibody (1:2000) and secondary HRP-conjugated anti-rabbit (1:1000) or Veriblot anti-IgG (1:1000) and processed as described for immunoblotting.

## Results

### N protein conservation and mutagenesis

**Fig 1:**
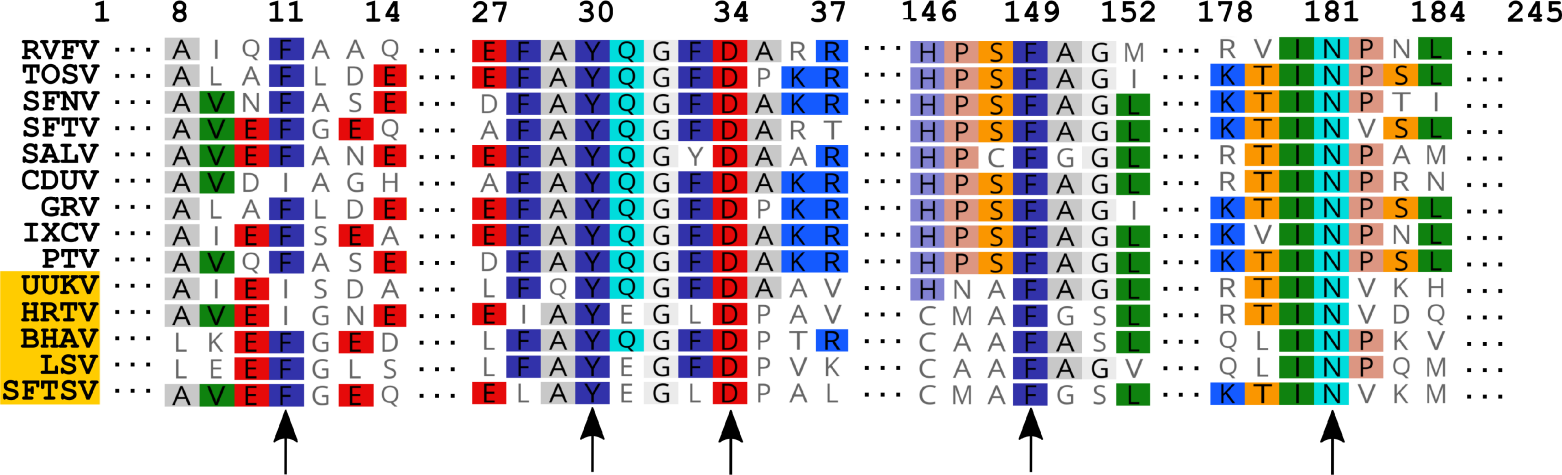
Alignment of Phlebovirus sequences highlighting point mutations. Rift Valley fever phlebovirus (RVFV), Toscana phlebovirus (TOSV), sandfly fever Naples phlebovirus (SFNV), sandfly fever Turkey phlebovirus (SFTV), Salehabad phlebovirus (SALV), Candiru virus (CDUV), Granada phlebovirus (GRV), Heartland phlebovirus (HRTV), Ixcanal phlebovirus (IXCV), Punta Toro phlebovirus (PTV), SFTS phlebovirus (SFTSV), Uukuniemi phlebovirus (UUKV), Lone Star phlebovirus (LSV), Bhanja phlebovirus (BHAV) sequences were aligned and conserved regions identified. The position of amino acids is relative to the RVFV nucleocapsid sequence. Tick-borne phleboviruses have been highlighted.

Fourteen *Phlebovirus* nucleoprotein sequences available from Genbank were aligned (Fig 1) and conserved residues were identified between all phleboviruses, between phleboviruses thought to be transmitted by an insect vector or phleboviruses transmitted by ticks. Of the conserved residues identified, five amino acids were selected for alanine substitution studies. Residues Y30 (previously analysed), D34, F149 and N181 in N protein sequences were found to be conserved across the whole *Phlebovirus* genus, derived from viruses transmitted by both insect and ticks. Residue F11, also previously analysed, is highly conserved between the mosquito-borne phleboviruses, with the exception of CDUV, but often substituted with an isoleucine within the UUKV-like tick-borne virus group.

**Fig 2:**
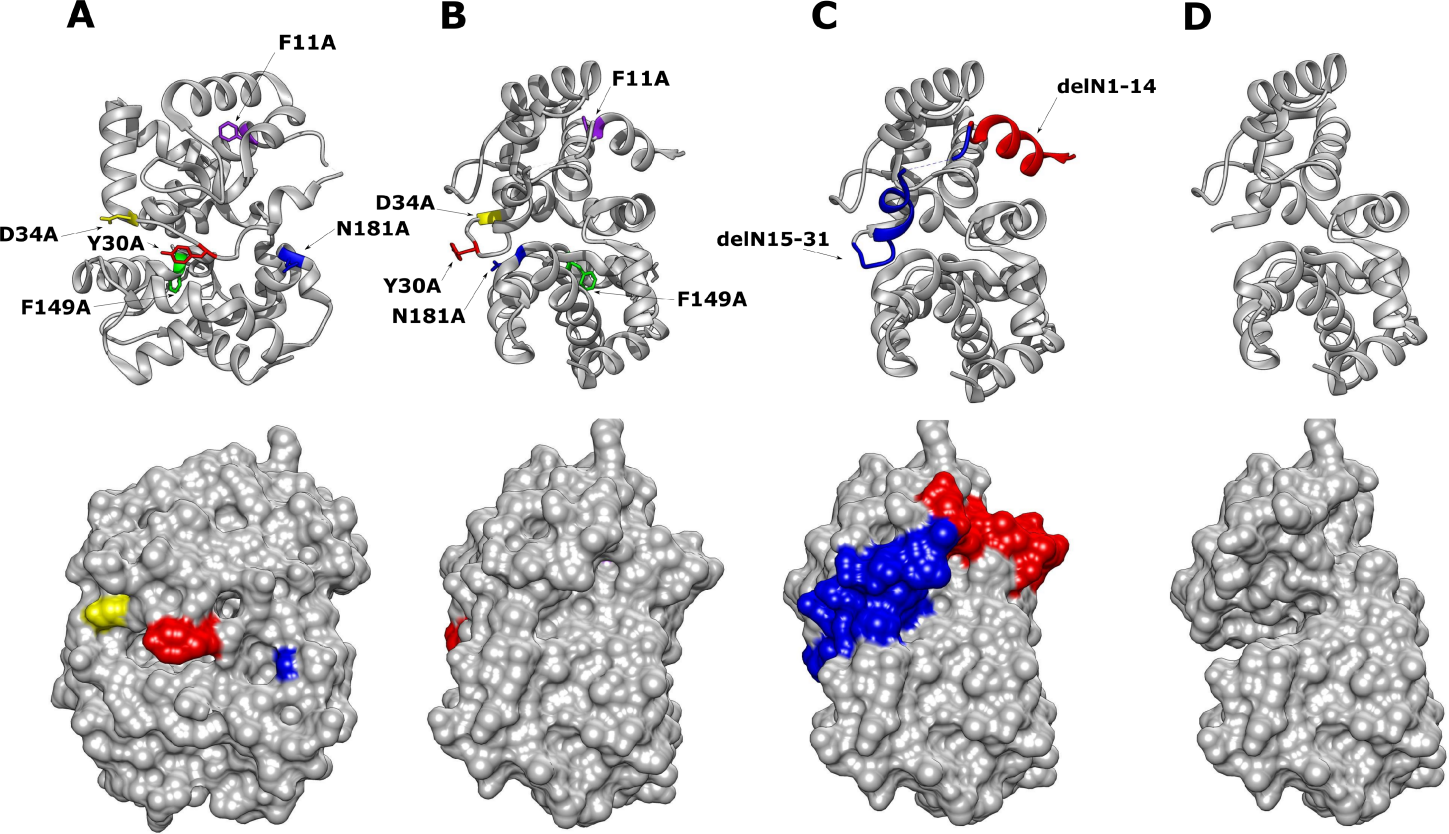
Chimera 3D Model of RVFV N protein monomeric structure with mutated residues highlighted (PDB: 3LYF) [18]. **(A)** RVFV N protein with highlighted point mutations (top image) and corresponding surface view (lower image). **(B)** Alternative orientation of the RVFV N protein point mutations at a 90-degree offset to (A). **(C)** N protein with highlighted N-terminal arm position 1–14 in red, 15–31 in blue. **(D)** Mutant RVFV N protein with the full delN1-31 arm removed and surface view.

The ambisense coding strategy of the *Phlebovirus* S segment results in differential expression of both the N protein and the non-structural protein NSs [9]. To evaluate the importance of these highly conserved N residues in isolation, without any interference of NSs on N protein or minigenome activity [22], an expression plasmid system containing only the N protein open reading frame (pTM1-N) was used over plasmids that express cDNA copies of the viral S segment RNA. The two N-arm mutants, a deletion of the amino acids 1–14 and 1–31, their location modelled in Fig 2, were also introduced to our panel of N protein mutants to examine the impact of N-N multimer interactions on N protein functions.

**Fig 3:**
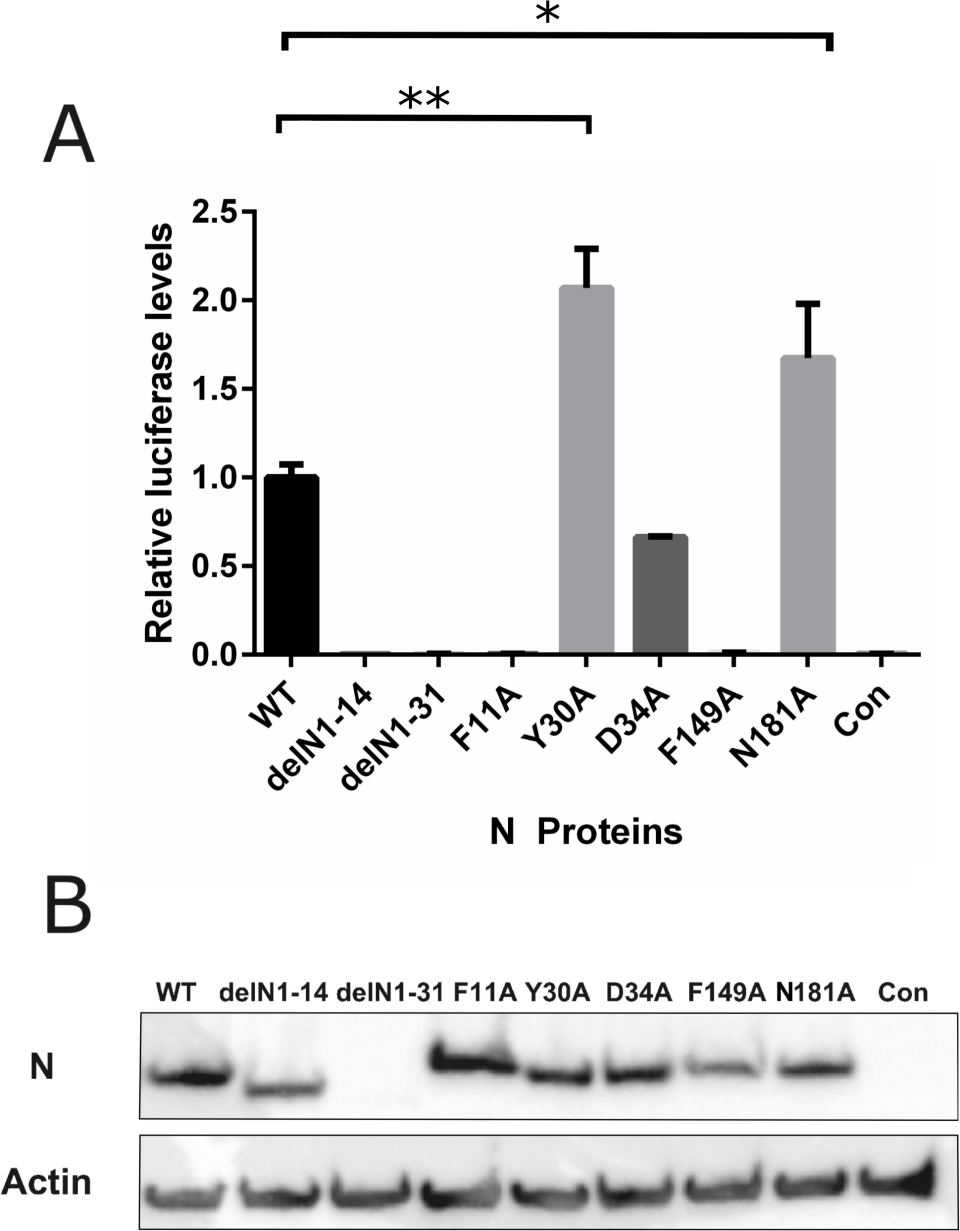
Effect of N protein mutations on RVFV-derived minigenome activity. BSR-T7/5 cells were transfected with pTM1-N (wildtype [WT] N, mutant N) or empty plasmid as negative control (Con) expressing-plasmids, pTM1-L, pTVT7-GM:hRen as well as pTM1-FF-Luc as a transfection control. **(A)** Values of triplicate experiments presented were calculated by dividing *Rluc* activity by *FFluc* luciferase activity to normalise differences in transfection efficiency; * denotes p<0.05, ** for p<0.001 using Student’s T-test. **(B)** Western blot of cell lysate probed with RVFV anti-N antibody (top panel) and anti-actin (bottom panel) as loading control.

Functional ribonucleoprotein complexes mediate the transcription and replication of the viral genome. This study aimed to assess the importance of the conserved residues identified from our phlebovirus N protein alignments. This was achieved using a previously described minigenome system, in which a virus genome segment analogue, where the viral coding sequence has been replaced with *Rluc* leaving the viral M segment UTRs intact, is transfected into T7 RNA polymerase expressing cells along with plasmids expressing the L protein and our N protein mutants [6, 24]. As seen in Fig 3A, the two N-arm mutants, delN1-14 and delN1-31 showed no activity in the minigenome system. Interestingly, the residues examined that were highly conserved within the *Phlebovirus* genus showed a range of activities. Mutants F11A and F149A had no replication activity, mutant D34 showed a decreased activity compared to wildtype (WT) N and mutants Y30A and N181A showed a significantly increased activity. Previous mutagenesis studies of BUNV N protein suggested that inconsistent protein expression levels may have significant effects on minigenome system activity [17]. As such a western blot for N protein was carried out using the cell lysates from the minigenome assay to control the expression levels of N protein. Expression levels of N protein were largely consistent between different transiently transfected N mutants (Fig 3B), except arm mutant delN1-31 which showed a greatly reduced expression level and was detected by using a BSR-T7/5 clone with higher T7 RNA polymerase activity produced during this project (S1 Fig).

### RNA binding properties of N protein mutants

**Fig 4:**
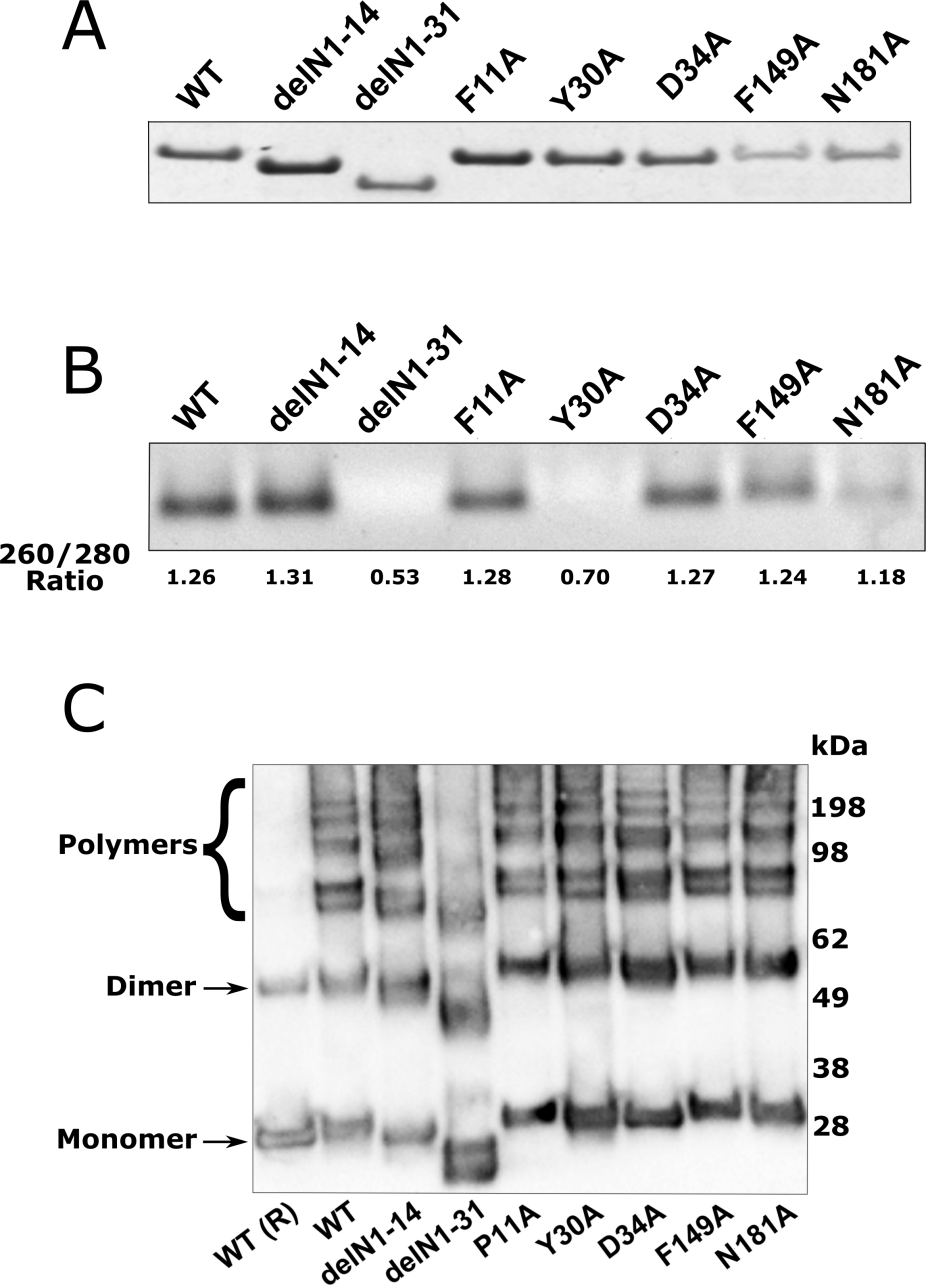
RVFV N protein binding properties as determined by binding of bacterial RNA during the purification process and multimerisation properties of N proteins. **(A)** InstantBlue stained gel of purified N proteins used in this study. **(B)** The *in vitro* RNA binding activity of WT and mutant N proteins as indicated was determined by observing the quantity of RNA dissociated after treatment with RNA loading buffer containing formamide representative of three repeats; 260/280 Ratios for this experiment are indicated. **(C)** Cross-linking was used to determine the formation of N multimers, and purified mutant proteins were cross-linked as described in Materials and methods, and analysed by western blot with RVFV anti-N antibodies. β-mercaptoethanol was added to the control (WT R) to reduce the di-sulfide bonds after multimerisation. This image is representative of three repeats.

RVFV N protein has two key functions; encapsidation of the viral RNA and multimerisation to allow for the formation of the viral RNPs [10, 18, 19]. To determine if the RNA binding capacity of the mutant panel was impaired, we carried out an RNA binding assay on purified mutant N proteins (Fig 4A). For this we used the viral N protein’s ability to bind non-specifically bacterial RNA, which occurs before and during the protein purification process, as shown previously [18, 25]. To determine whether this function was impaired, RNA was dissociated from the purified N protein using formamide-containing RNA loading buffer [25]. As shown in Fig 4B, RNA binding activity was observed in the majority of mutant N proteins examined. However, neither the arm mutant delN1-31 nor mutant Y30A had detectable RNA bound to mutant N protein when purified, which was consistent with our measurement of 260/280 ratio for all these proteins.

### Multimerisation properties of N proteins

The N protein of the Smithburn vaccine strain of RVFV was shown to form higher order structures: tetramers, pentamers and hexamers in multiples of the 27 kDA monomer [10]. Here we assessed whether the minigenome activities observed previously resulted from changes in the multimerisation. The multimerisation properties of the mutant N proteins panel were determined through a DSP crosslinking assay on purified N protein from a bacterial expression system. Only the delN1-31 mutant showed a reduced capacity to multimerise and all other mutants showed multimerisation capacity similar to WT N protein (Fig 4C).

### Interactions of N and L proteins

**Fig 5:**
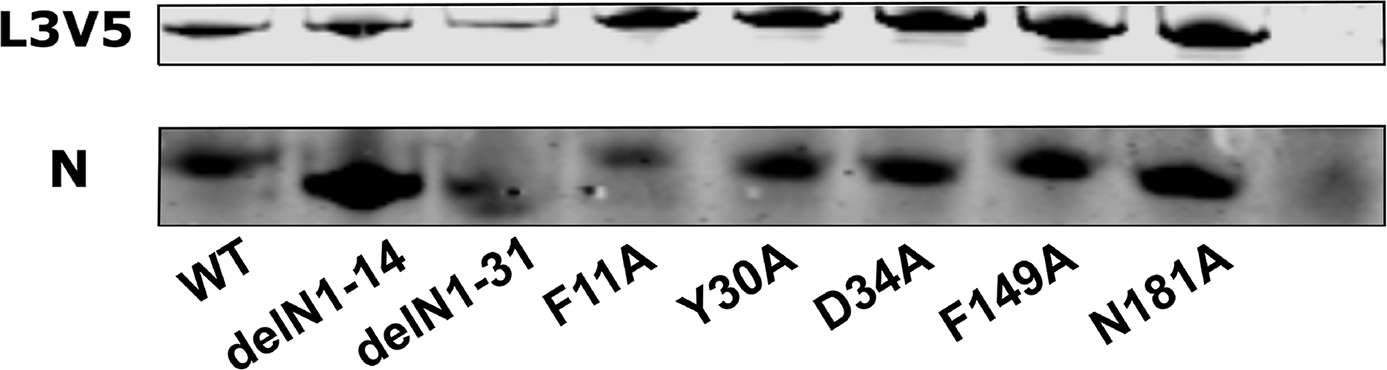
Interaction of L3V5 viral polymerase with WT and mutant N proteins. BSR-T7/5 CL21 cells were transfected with mutant or WT pTM1-N and pTM1-L3V5. Negative control was transfected pTM1-N without pTM1-L3V5 (Con). After 24 hours, cells were lysed and the lysate was applied to magnetic beads carrying V5 antibody. The bound L protein was dissociated from the beads and analysed by western blot. The proteins were fractionated on a 4–12% Bis-Tris plus (Novex) gel and transferred to a nitrocellulose membrane. Subsequently the blot was probed with anti-V5 antibody and RVFV anti-N antibodies and visualised using LI-COR. This image is representative of three repeats.

The ability for the N protein to interact with the viral L protein is important for the formation of replication-active RNP complexes. By using a construct expressing a V5-tagged L protein (L3V5) [22], we were able to perform co-immunoprecipitation (co-IP) studies to assess the interaction of L protein with our panel of mutant N proteins. The co-IP was performed under RNase conditions to reduce the effect of bound RNA influencing the interaction, though RNA bound within N protein prior to co-IP would not be removed in these conditions. Following co-IP with an anti-V5 antibody, western blotting with anti-N and anti-V5 sera was performed to identify if N protein co-immunoprecipitated with the L protein. The mutants found to be functional in the minigenome system (Y30A, D34A and N181A) interacted with the L protein. Intriguingly, N mutants’ delN1-14, delN1-31 and F11A and F149A could also bind to the L protein despite not being active in the minigenome assays (Fig 5). This suggested that any functional deficiencies observed are not due to absence of direct interactions between the replication complex proteins, though L polymerase processivity could be affected.

### Impact of N mutations on packaging and virus-like particle (VLP) formation

**Fig 6:**
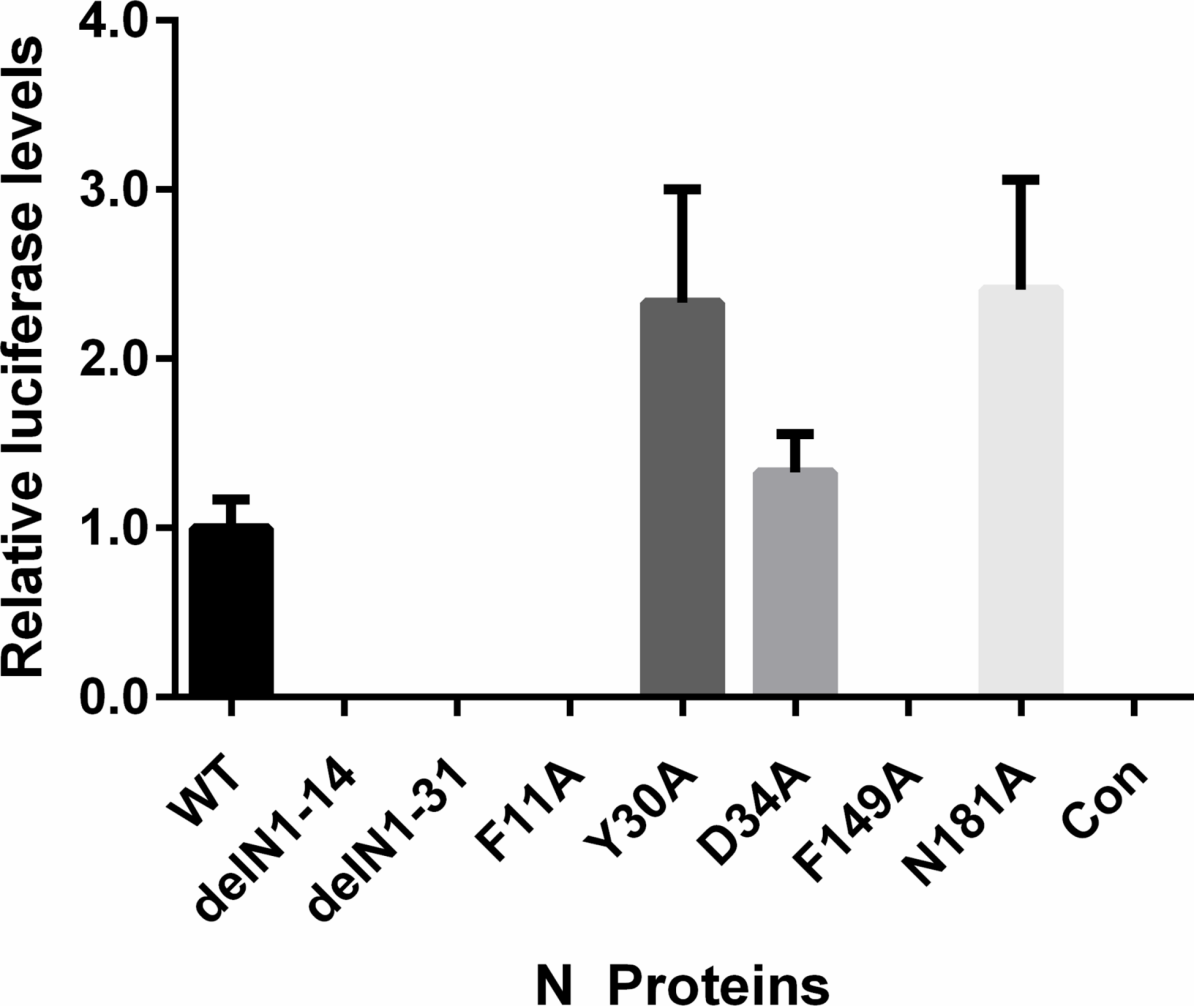
Activity of RVFV N protein mutants in a VLP assay. BSR-T7/5 CL21 cells were transfected with pTM1-L, pTVT7-GM:hRen, pTM1-M, pTM1-FF-Luc and mutant or WT pTM1-N as described. Negative control cells were transfected with full complement of plasmids without pTM1-N (Con). After 48 hours, the supernatant was harvested and Benzonase treated. The treated supernatant was applied to BSR-T7/5 CL21 cells transfected 24 hours prior with pTM1-N and pTM1-L. *Rluc* activity was measured at 24 h post-infection. Experiments were carried out in triplicate and repeated three times.

Amongst other functions, RVFV N protein has been implicated in an interaction with the first 30 amino acids of the cytoplasmic tail of the viral glycoprotein Gn to mediate efficient packaging of viral RNP complexes into newly forming virus particles [26]. To determine if any of the mutated amino acids of N protein abrogated packaging, we employed an assay based on packaging of a minigenome segment into VLPs. Briefly, BSR-T7/5 CL21 cells were transfected with a RVFV M segment based humanised *Rluc* minigenome-encoding plasmid (pTVT7-GM:hRen), plasmids encoding the L protein and WT N protein or one of the mutant N proteins, and a *FFluc* expressing plasmid as a transfection control. The transfection mixes were also supplemented with expression plasmid encoding the RVFV M segment glycoprotein precursor. At 48 h post transfection, the cell culture media of the donor cells (cells used to generate VLPs) was clarified by centrifugation and nuclease treated to prevent plasmid carry over. Subsequently the nuclease-treated VLP containing supernatant was used to inoculate recipient BSR-T7/5 CL21 cells transiently transfected with plasmids expressing the RVFV L protein and WT N protein. In the case of functional packaging of the minigenome segment into virions, the encapsidated pTVT7-GM:hRen is delivered to the recipient cell by the VLP where it is replicated, transcribed and translated resulting in *Rluc* expression (Fig 6). The VLP assay data corroborated with the minigenome data presented in Fig 3A, showing that only mutants Y30A, D34A and N181A that were functional in the minigenome system also could form functional, minigenome containing VLPs.

## Discussion

In this study, we sought to gain a greater understanding of the biology and functions of the RVFV N protein through the identification of amino acids that are highly conserved among phleboviruses and that have yet unknown functions. RVFV N is a very highly conserved protein in nature as evidenced by multiple sequence alignment of different isolates, and as such there is a higher propensity of conserved residues to confer significant function. Of the conserved residues identified, both F11A and F149A had unidentified roles which appeared essential for the replication or transcription of viral RNPs and/or formation of RNP complexes. The F149A mutant showed no activity in minigenome assay and has not been previously described in the literature. We hypothesise that due to this amino acid being surface exposed within a protein cleft found in the N protein structure, it may be involved in the binding of host factors required for these processes. A F11G mutant has previously been shown to be involved in loss of N-N dimer formation, by a different assay system and using GST-fused N protein, through the potential misfolding of the N-terminal region and disruption of the N-N interaction [20]. Interestingly here we show that purified F11A mutant protein of RVFV N was still able to form dimers and higher order structures within our multimerisation assay system. This discrepancy may be explained by the glycine substitution resulting in a less stable structure. While it is clear that the F11 residue is essential for N protein function, it may be involved in other interactions relevant for replication other than the ascribed N-N dimer formation or interactions. These could involve, for example, processivity of RNA synthesis or specific interactions with host factors that are required for virus replication.

We also identified two residues, Y30 and N181, which showed increased activity in the minigenome system (Fig 3). The residue N181 has no previously described function, but the N181A mutant was also able to form functional virions in the VLP assay system (Fig 6). Y30A was considered to be essential in a previous study [19].

Despite evidence that Y30 is an essential residue within the N-terminal arm, involved in the multimerisation of N protein and the base stacking of RNA within the RNA binding groove, the Y30A mutant used in this study only appeared to have reduced RNA binding capacity but retained the ability to form multimers. This corroborated with a previous study indicating that the Y30A mutant did not disrupt N-N interactions [20]. This reduced RNA binding capacity of Y30A did not negatively affect the overall expression of the minigenome reporter gene or packaging into VLPs. There may be an inverse relationship between minigenome activity and RNA binding, it has been proposed that N may non-specifically bind mRNA preventing translation however luciferase transfection control levels showed no significant change in the presence of N (S2 Fig). A mutational analysis of UUKV, also of the *Phlebovirus* genus, observed that the Y30A mutant still had activity in a minigenome reporter assay [27]. However, in the Katz et al. study, further analysis of RNA binding was not carried out. It is possible that the reduced RNA binding capacity may positively affect RNA replication at the loss of protection from degradation, however this requires further study. The formation of functional VLPs also shows that the Y30A mutant still interacts with the Gn glycoprotein to successfully package the reporter construct.

Both the delN1-14 and delN1-31 N protein mutants showed no activity in the minigenome assay. UUKV N N-terminal mutants also showed no activity in minigenome-based assays analysed by the detection of CAT signal. These data support the hypothesis that the N-terminal arm of phleboviral N proteins are functionally essential [27]. However, the delN1-14 mutant N protein was still able to form higher order multimers and bound RNA. Based on our RNA-binding and multimerisation analysis data, it suggests that N protein is still able to efficiently multimerise and bind RNA without the 1^st^ helix of its N-arm, these functions are impaired if the second helix of the arm is also removed. However, if the whole arm was removed, oligomeric structures were still formed, although at a very low efficiency, suggesting that there is another mechanism or region involved in N-N binding. Comparatively, a delN1-19 mutant in UUKV N showed approximately 25% N-N binding capacity indicating that binding was still occurring at reduced levels [27]. It is possible that the loss of function is due to the impaired ability of the RNA-dependent RNA-polymerase L to track along the viral RNP and/or due to the formation of N protein monomers in an incorrect configuration. The lack of activity found in minigenome assays with delN1-31 N mutant is likely due to its inability to form higher order structures and bind viral RNA, though the ability for delN1-31 mutant to interact with L also indicates that the N-terminal arm is not involved in the direct interaction between RNA-dependent RNA polymerase and viral nucleocapsid protein.

In summary, this study expands the number of known essential residues of RVFV N protein that are conserved across phleboviruses, while also informing that other residues may have additional or different functions than previously documented in RVFV. The availability of protein residue information is an important resource for further studies into the functions and potential therapeutic targets of RVFV N protein. The conserved nature of these residues in N proteins also may indicate conserved functions or interactions across the whole *Phlebovirus* genus and thus provide important information beyond what we currently understand for RVFV.

## Supporting information

S1 Fig Relative expression levels of delN1-31 mutant. Western blot of pTM1 N and delN1-31 mutant expression in BSR-T7/5 CL21 cells. Extracts of four times more cells transfected with delN1-31 were loaded for expression analysis.
(TIF)

S2 Fig Effect of N protein on luciferase expression. Firefly luciferase (*FFluc*) values for the experiment described in Fig 3. Values of triplicate experiments presented. Student’s T-test was performed and determined there was no significance between results.
(TIF)
