# Supplementary figures and images for: Mutational analysis of Rift Valley fever phlebovirus nucleocapsid protein indicates novel conserved, functional amino acids
Source: PLoS Negl Trop Dis. 2017 Dec 21;11(12):e0006155. doi: 10.1371/journal.pntd.0006155 (PMC5764413; doi:10.1371/journal.pntd.0006155)

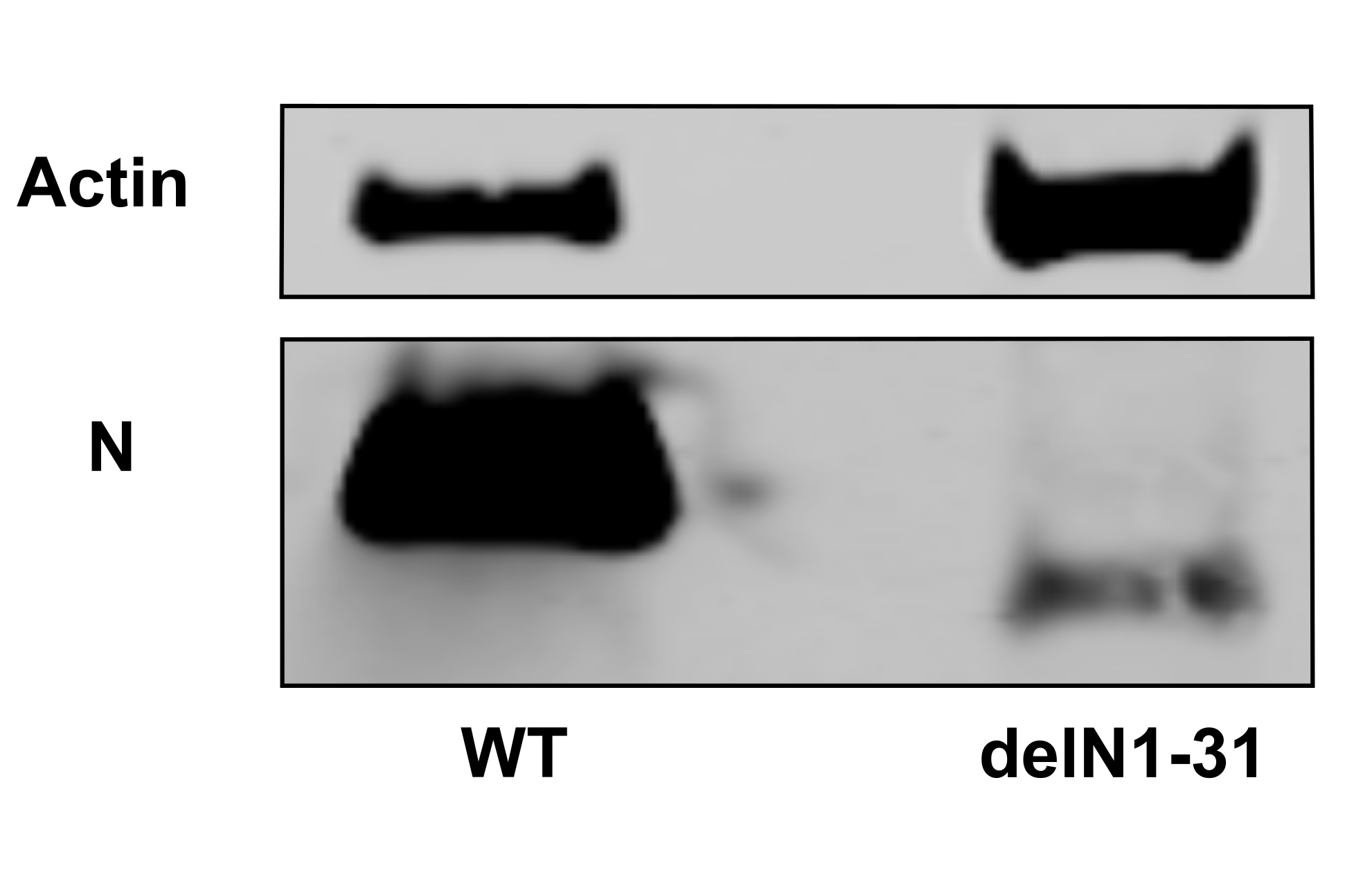

Supplement: S1 Fig — Western blot of pTM1 N and delN1-31 mutant expression in BSR-T7/5 CL21 cells. Extracts of four times more cells transfected with delN1-31 were loaded for expression analysis. (TIF) [file pntd.0006155.s001.tif]

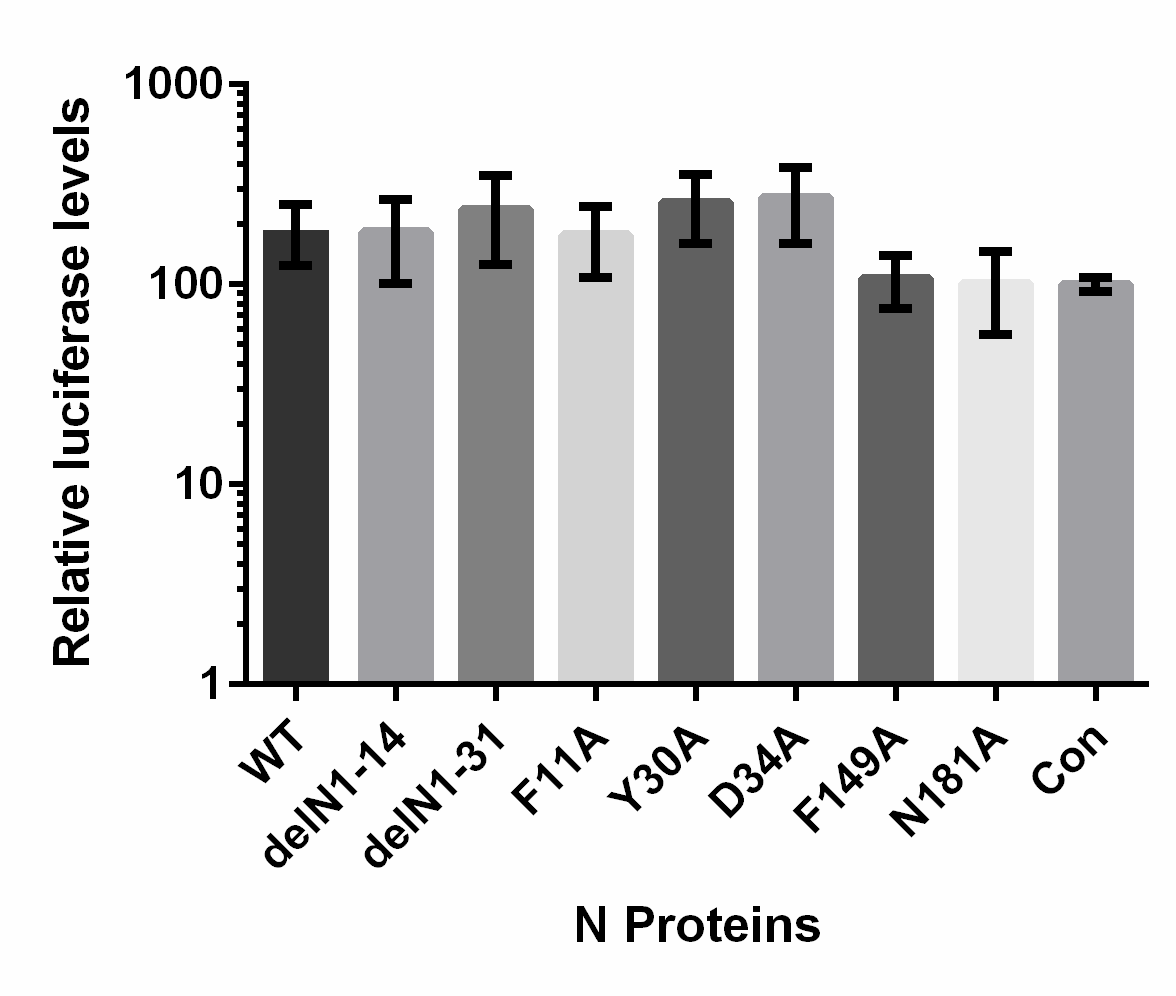

Supplement: S2 Fig — Firefly luciferase (FFluc) values for the experiment described in Fig 3. Values of triplicate experiments presented. Student’s T-test was performed and determined there was no significance between results. (TIF) [file pntd.0006155.s002.tif]
